# The Updated NICE Guidelines: Cardiac CT as the First-Line Test for Coronary Artery Disease

**DOI:** 10.1007/s12410-017-9412-6

**Published:** 2017-03-27

**Authors:** Alastair J. Moss, Michelle C. Williams, David E. Newby, Edward D. Nicol

**Affiliations:** 1 University of Edinburgh/British Heart Foundation Centre for Cardiovascular Science, Chancellor’s Building, 49 Little France Crescent, Edinburgh, EH16 4SB UK; 2 Department of Cardiology, Royal Brompton Hospital and Harefield NHS Trust, Sydney Street, London, SW3 6NP UK

**Keywords:** Coronary computed tomography angiography, Chest pain, Angina, Coronary artery disease

## Abstract

**Purpose of Review:**

Cost-effective care pathways are integral to delivering sustainable healthcare programmes. Due to the overestimation of coronary artery disease using traditional risk tables, non-invasive testing has been utilised to improve risk stratification and initiate appropriate management to reduce the dependence on invasive investigations. In line with recent technological improvements, cardiac CT is a modality that offers a detailed anatomical assessment of coronary artery disease comparable to invasive coronary angiography.

**Recent Findings:**

The recent publication of the National Institute for Health and Care Excellences (NICE) Clinical Guideline 95 update assesses the performance and cost utility of different non-invasive imaging strategies in patients presenting with suspected anginal chest pain. The low cost and high sensitivity of cardiac CT makes it the non-invasive test of choice in the evaluation of stable angina. This has now been ratified in national guidelines with NICE recommending cardiac CT as the first-line investigation for all patients presenting with chest pain due to suspected coronary artery disease. Additionally, randomised controlled trials have demonstrated that cardiac CT improves diagnostic certainty when incorporated into chest pain pathways.

**Summary:**

NICE recommend cardiac CT as the first-line test for the evaluation of stable coronary artery disease in chest pain pathways.

## Introduction

Despite decades of investment in the delivery of effective treatment, cardiovascular disease remains responsible for the vast majority of deaths worldwide. Even prior to the recent fluctuations in the global financial climate, many modern healthcare economies had sharpened their focus on delivering healthcare that is both clinical and cost effective. Ensuring cost-effective and efficacious treatment is central to maintaining sustainable healthcare programmes as populations increase in age and access healthcare in greater numbers. In the UK, the National Institute for Health and Care Excellence (NICE) routinely reviews clinical evidence and publishes advice and recommendations based on the combination of clinical efficacy and cost effectiveness of both therapies and technologies. Established in 1997, it is now enshrined in UK law to ensure patients cared for in the English National Health Service (NHS) gain benefits from cost-effective measures, and that NHS resources are optimised.

Guidelines on the investigation and management of coronary artery disease (CAD) have evolved significantly over the past two decades. In the early 1990s, Braunwald’s *Quick Reference Guide for Clinicians* recommended patients were offered treatment based on their pre-test probability of angina, and a clear history of anginal chest pain was a pre-requisite for stratifying which patients required downstream investigation [1]. Non-invasive testing (predominantly exercise-EKG) was reserved for identifying high-risk patient groups that may benefit from additional medical therapy and coronary revascularisation.

Since Braunwald’s early publication, the expansion of multi-modality cardiac imaging has generated a number of different testing strategies with improved sensitivity for diagnosing CAD that have been incorporated into national [2] and international guidelines [3, 4]. Due to the risk of potential complications following the onset of chest pain symptoms, clinical practice now focuses on the rapid clinical assessment of patients with suspected angina to select out high-risk individuals [5].

Prior to being updated in November 2016, the UK NICE recommendations on the investigation of stable chest pain involved eliciting a careful history of symptoms and estimation of the pre-test likelihood (PTL) of significant CAD using a modified Diamond-Forrester model [6] to select the appropriate non-invasive test for improving diagnostic certainty of CAD [2]. Only those with typical and atypical angina symptoms with a >10% PTL of obstructive CAD were recommended for cardiac investigations to confirm the diagnosis angina secondary to obstructive CAD. In patients with a PTL <10 or >90%, no cardiac investigation was recommended. If patients had a PTL >90%, invasive coronary angiography was reserved for those who remained symptomatic despite optimal medical therapy for stable angina or in asymptomatic cases where there was a suspicion of prognostically significant CAD that may benefit from coronary revascularisation. If the PTL was low (10–29%), a Coronary Artery Calcium Score (CACS) was the recommended first-line investigation with subsequent CT coronary angiography (CTCA) if the calcium score was between 1 and 400 Agatston Units. If the PTL was intermediate (30–60%), non-invasive functional imaging was recommended as the first-line investigation, with invasive coronary angiography (ICA) recommended for those with a high PTL (61–90%). Adherence to this guideline provided considerable cost savings, namely from a reduction in the number of expensive ICAs in spite of a more costly initial non-invasive testing strategy [7].

Several concerns were subsequently raised about the 2010 NICE guidelines and many were fundamental to the recent revision. First, the reduction in the prevalence of CAD secondary to the increased use of therapy for risk factor modification led to the modified Diamond-Forrester model significantly overestimating the frequency of significant CAD, particularly in women [8]. Clearly, the use of a PTL model is only valid in risk stratification if it identifies and groups patients accurately and reproducibly.

Second, there was concern about the minimal use of anatomical assessment of CAD that was limited to CACS and ICA in those with the lowest and highest PTL. Moreover, the evidence base for CACS was based on data from asymptomatic patients, not those with symptoms [9]. As such, the appropriateness of CACS as the first-line investigation with subsequent CTCA reserved for those with a CACS between 1 and 400 AU was challenged [10] with the UK Royal College of Radiologists stating CTCA would be reasonable to perform in those with a CACS of 0.

Third, even in 2010, CTCA assessment was likely to be beneficial in those with both low and intermediate PTL [11] and functional imaging for all intermediate PTL would undoubtedly lead to both false-positive and false-negative results based on sensitivity and specificity of these techniques [12, 13].

Thus, the updated 2016 NICE guideline is notable for its removal of the pre-test probability model and the use of CTCA as the first-line investigation in all patients with atypical or typical angina symptoms or those who are asymptomatic with suggested EKG changes for ischemia [14]. It has been argued that service provision for those with stable CAD should be tailored towards the needs of the population to optimise cost-efficacy in a testing strategy that is most applicable to the low-intermediate PTL group of patients, as this would cover the majority of patients presenting to chest pain clinics [15]. Thus, a test with a high negative predictive value has great merit in the assessment of patients with suspected angina due to CAD.

Is this anatomical-guided strategy supported by the evidence? Historically, ICA has been the definitive test to confirm of the presence of CAD against which all other non-invasive tests have been validated. However, ICA is the most expensive diagnostic investigation and, importantly, exposes individuals to the highest risk of procedural complications [14]. In the 2016 update, NICE determined the clinical effectiveness of the non-invasive modalities against a “gold standard” of 50% stenosis on ICA, the same methodology that was used in the previous guidelines issued in 2010. Confirming the presence of a coronary artery luminal stenosis of 50% or greater is only part of the diagnostic assessment. Confirmation of inducible myocardial ischemia has been the principal goal prior to the initiation of appropriate of secondary prevention treatment and coronary revascularisation. In the NICE guidelines, functional imaging is still recommended for those who have significant CAD or equivocal findings on CTCA. It should also be noted however that whilst non-invasive testing can assess multiple levels of the ischemia cascade, it is ultimately the confirmation of the anatomical burden of CAD which determines an individual’s future risk of cardiac events [16].

## Clinical Effectiveness of CTCA against ICA

As part of the 2016 update, NICE evaluated the diagnostic accuracy of the main non-invasive testing strategies against the presence of obstructive coronary artery luminal stenosis on ICA. Established stress testing modalities using dobutamine or exercise stress echocardiography (DSE/ExSE), myocardial perfusion scintigraphy (MPS)–single photon emission computed tomography (SPECT), and cardiac magnetic resonance imaging (CMR) have comparable levels of diagnostic accuracy, which is reflected by the relatively few novel publications in this field. Conversely, the evolution of computed tomography (CT) has resulted in technological developments providing increased volume coverage and shorter gantry rotation times that significantly improve the diagnostic utility at much reduced levels of radiation exposure. The enhanced spatial and temporal resolution of modern cardiac CT scanners offer an extremely high sensitivity (0.96, 95% CI 0.94–0.97) for detecting the presence of coronary artery plaque when compared to ICA. The majority of the studies included in the NICE meta-analysis were composed of populations with a higher prevalence of coronary artery disease than is often found in the unselected population that are referred to cardiology clinics for assessment. This may account for the wider variation seen in the specificity of cardiac CT (0.79, 95% CI 0.72–0.84), albeit no significant bias was observed following sensitivity analysis (0.79, 95% CI 0.73–0.85, I2 79%). It is no surprise that when compared with ICA, an anatomical assessment using cardiac CT outperforms all other stress testing modalities. However, of greater importance is the ability to avoid more costly and potentially harmful invasive investigation (non-fatal complication 74 per 10,000 (ICA) versus 3.2 per 10,000 (CTCA) [14].

## Cost Effectiveness of CTCA

**Fig. 1:**
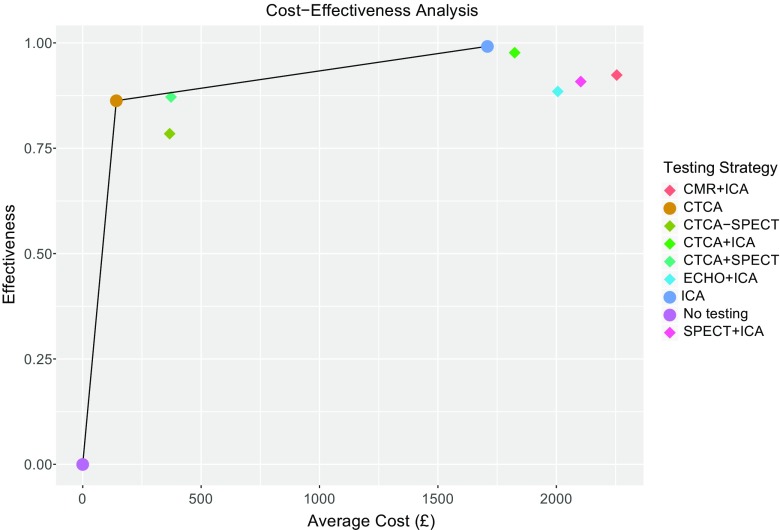
NICE cost-effectiveness analysis of diagnostic tests in 45% pre-test likelihood population. [14]. The figure plots the average proportion of correct diagnoses (effectiveness) versus the average cost (£ sterling) of each testing strategy. A first-line testing strategy using CT coronary angiography has the lowest cost per correct diagnosis of coronary artery disease. The cost-effectiveness frontier is represented by a *line* connecting no testing, CT coronary angiography, and invasive coronary angiography. All other testing strategies lie beneath this line and have fewer correct diagnoses at a higher cost

NICE’s cost utility analysis of a diagnostic test aims to calculate the incremental cost and benefit of care pathways using different investigation strategies. The health economics of care pathways have come under greater scrutiny due to increasing financial pressures on healthcare providers. Only two studies have been published that reflect both the UK costs of investigation for CAD and measurable health benefit by either quality-adjusted life year or correct diagnosis [17, 18]. Genders et al. investigated 16 different diagnostic strategies involving cardiac CT, CMR, echocardiography and MPS-SPECT. A lifetime time horizon and Markov state-transition model were applied to a simulated population of 60-year-olds with no history of coronary artery disease. By applying costings from 2011, DSE was the most cost-effective test. The NICE cost utility analysis used tariffs derived from NHS reference costs (CTA £122.11, DSE £271.31, MPS-SPECT £367.29, CMR £515, ICA £1684.71) and cardiac CT was, by far, the lowest-cost test per correct diagnosis due to the low cost of the test and high sensitivity and low probability of fatal or non-fatal complication. Figure 1 demonstrates graphically the cost and clinical effectiveness of various strategies in a simple to understand format. Interestingly, economic modelling determined that only if the cost of cardiac CT tripled would it cease to be the least cost-effective initial investigation, and even then the next most cost-effective strategy was a combination of CTCA with DSE (cost at which CTCA is no longer cost-effective, 20% pre-test probability £394.95, 45% pre-test probability £494.84, 75% pre-test probability £710.32).

NICE has calculated that the use of CTCA as a first-line investigation will generate annual savings of £16 million in England alone, by prompt exclusion of significant CAD and more effectively use of NHS resources. [19] Modelling for US tariffs has not been performed, but cost components based on the cost utility analysis of the PROMISE study compare favourably [20] and the UK modelling is likely to be broadly applicable in other healthcare settings.

## Beyond the NICE Guidelines

Whilst the UK NICE guidelines give a clear picture as to the most clinically and cost-effective modality based on a 50% stenosis on ICA as a gold standard, there are additional arguments for the use of CTCA as a first-line investigation in this population. The ideal diagnostic test for CAD should influence downstream decision-making to reduce cardiac death and non-fatal myocardial infarction. To facilitate a reduction in cardiac events, the first-line diagnostic test should have a high level of diagnostic accuracy, the ability to better risk stratify individuals into different treatment regimens and be integrated into a cost-effective clinical pathway.

Until recently, most studies that have compared different stress imaging modalities to detect obstructive CAD have found no definitive evidence to recommend the superiority of one diagnostic test over another [21, 22]. Diagnostic evaluation using functional testing has received significant criticism due to the high rate of false-positive results. Registry data have highlighted the low prevalence of obstructive CAD following elective ICA, despite prior stratification with functional testing to identify prognostically significant myocardial ischemia [23, 24]. The low diagnostic yield of actionable CAD using functional testing has generated a debate as to which test is best placed to serve as a ‘gatekeeper’ to ICA. The recent publication of Clinical Evaluation of Magnetic Resonance Imaging in Coronary Artery Disease 2 (CE-MARC-2) supported the rationale for non-invasive testing prior to ICA, owing to risk models overestimating the presence of obstructive CAD as previously discussed [21]. With the multitude of non-invasive tests available, ICA is only rarely required to confirm the diagnosis of obstructive CAD and should be reserved for those likely to have coronary intervention. At the time of the NICE guideline review, two ‘test-and-treat’ multicentre randomised control trials were published that provided insight into whether CTCA could be incorporated into chest pain care pathways to improve diagnostic accuracy and risk stratification of coronary artery disease [22, 25]. Both trials were discussed in the update of the NICE recommendations, as they have laid new ground for evaluating the efficacy of non-invasive imaging tests, namely in determining whether their incorporation into a care pathway actually confers benefit to patients. They also offer an opportunity to assess the merits of using an anatomical-guided strategy in chest pain pathways in a combined cohort of over 14,000 patients (Table 1).

**Table 1:** Anatomical-guided strategy using cardiac CT in a combined cohort of over 14,000 patients

SCOT-HEART and PROMISE trials				
Study	SCOT-HEART		PROMISE	
Population	4146 patients Chest pain 100% Typical angina 35% Previous CAD 9%		10,003 patients Chest pain 73% Typical angina 12% No previous CAD	
Randomisation	1:1		1:1	
Intervention	CTCA in addition to standard care		CTCA versus functional test	
Control	Standard care Nuclear stress imaging (9%) Stress echocardiography (1%) Exercise EKG (85%)		Functional test Nuclear stress imaging (67.3%) Stress echocardiography (22.5%) Exercise EKG (10.2%)	
Primary outcome	Certainty of diagnosis of angina due to coronary heart disease at 6 weeks		All cause mortality, non-fatal MI, hospitalisation for unstable angina, major procedural complications	
	CTCA (*n* = 2073)	Standard care (*n* = 2073)	CTCA (*n* = 4996)	Functional test (*n* = 5007)
CAD, >50% stenosis (*n*)	42% (752)	–	10.3% (517)	–
All-cause death/non-fatal MI^a^ (n)	1.9% (39)	2.7% (55)	2.1% (104)	2.2% (112)
Cardiac death/non-fatal MI^a b^	1.3% (26)	2.0% (42)	–	–
Non-fatal MI^a^ (*n*)	1.1% (22)	1.7% (35)	0.6% (30)	0.8% (40)
Revascularisation (*n*)	11.2% (233)	9.7% (201)	6.2% (311)	3.2% (158)
Cardiac death/non-fatal MI in revascularisation group (*n*)	8% (18)	14% (28)	–	–

^a^SCOT-HEART median follow-up 1.7 years, PROMISE median follow-up 2 years

^b^SCOT-HEART hazard ratio 0.62, 95% confidence interval 0.38–1.01, *p* = 0.0527

The Prospective Multicenter Imaging Study for Evaluation of chest pain (PROMISE) trial was a pragmatic trial that recruited a large cohort from USA and Canadian centres to determine whether an initial assessment of suspected stable CAD using CTCA reduces major adverse cardiovascular events [22]. There was no improvement in death, myocardial infarction or major procedural complication after a median of 2-years of follow-up when compared with a functional-guided strategy. The Scottish Computed Tomography of the HEART (SCOT-HEART) trial recruited patients referred for recent onset chest pain to cardiology clinics with suspected angina [25]. The trial population reflected standard UK practice, all of whom presented with chest pain and one third reported typical angina symptoms. In the CTCA arm, a higher rate of obstructive CAD was reported compared with the PROMISE trial (Table 1), and, of note, there was a borderline but non-significant reduction in cardiac death and myocardial infarction (hazard ratio 0.62, 95% confidence interval 0.38–1.01, *p* = 0.0527). Subsequent post hoc landmark analysis from the time of clinical intervention (at 50 days), namely the point when clinicians reviewed the test result and dispensed preventive medical therapy, was associated with a halving of the rate of cardiac death and myocardial infarction (hazard ratio 0.50, 95% confidence interval 0.28–0.88, *p* = 0.020) [26]. This signal is consistent with observational data from the CONFIRM registry showing that initiation of statin therapy in individuals with subclinical atherosclerosis confers a survival benefit [27].

## Challenges to the Implementation of the Updated NICE Guideline

The publication of the updated NICE guideline CG95 poses some major challenges to the cardiovascular imaging community. First, a significant increase in the availability of CTCA is required for UK centres to comply with the updated NICE recommendations. An estimate by the British Society of Cardiovascular Imaging/British Society of Cardiovascular CT (BSCI/BSCCT) is that a 700% increase in cardiac CT will be required across the UK [28]. In order to fulfil this service commitment, substantial investment in CT technology and training will be required. The UK has a relatively low number of CT scanners per head of population compared to other countries in Europe and the USA [29], and whilst centres that already perform a high volume of cardiac CT will have fewer delivery challenges than the low volume centres, there are currently no centres in the UK which are meeting the required targets for compliant delivery of cardiac CT imaging.

Second, there will also be implications for functional imaging modalities which are more difficult to define. Concerns involve the potential disinvestment in stress imaging services in favour of CT imaging. Whilst a second-line stress test may be required in a proportion of patients who undergo CTCA, it is unknown how this will affect the service utilisation of functional imaging. The increased use of CTCA will generate greater numbers of positive and equivocal CTCA and therefore the requirement for functional imaging will likely not decline significantly.

Third, in addition to increasing the volume of cardiac CT that is performed, it is essential that quality is maintained in order to maintain the high diagnostic accuracy of CTCA. This includes optimisation of image quality, providing informative reports of studies and minimising radiation dose exposure. Guidelines for the optimal acquisition of cardiac CT have been published by national and international groups, and these should be followed in order to optimise image quality in cardiac CT [30, 31]. Guidelines are also available for the structured reporting of cardiac CT which may help with the communication of results to clinicians and patients [32]. This may also assist in the rationalisation of downstream investigations and prevent a reactionary increase in the use of invasive coronary angiography when cardiac CT identifies non-obstructive CAD.

Finally, the emergence of cardiac CT has correlated with a debate on the revision of atherosclerotic coronary artery disease classification [33]. Categorising CAD on the basis of stenosis severity alone fails to account for the continuum of risk associated with non-obstructive atherosclerotic plaque. Indeed, improved risk stratification of future cardiac events may be achieved by identifying features of vulnerability in metabolically active plaques rather than relying on luminal narrowing in isolation [34]. Whilst currently there is no clear guidance on how to manage non-obstructive CAD, there is growing recognition that this is currently an unmet public health problem, particularly in women [35].

## Conclusion

The update to the UK NICE guidelines recommends that cardiac CT is the first-line investigation for patients presenting with new-onset chest pain due to suspected CAD. This guideline is based primarily on the diagnostic accuracy and cost effectiveness of this strategy, using ICA stenosis as the gold-standard. In addition, the inability of cardiovascular risk assessment to adequately differentiate between groups of patients means that this has been removed from the updated NICE recommendations. An anatomical investigation strategy, such as proposed in the 2016 NICE guidelines, is supported by recent large-scale randomised controlled trials using cardiac CT in both the UK and USA. The implementation of these guidelines requires a large increase in the number of CT scans that are performed; however, the projected cost savings of $20 million per annum, in England alone, mean that the short-term investment is likely to be justified.

## Abbreviations

CACS: Coronary artery calcium score
CAD: Coronary artery disease
CTCA: Computed tomography coronary angiography
CMR: Cardiac magnetic resonance
DSE: Dobutamine stress echocardiography
EKG: Electrocardiogram
ExSE: Exercise stress echocardiography
ICA: invasive coronary angiography
MI: Myocardial infarction
MPS–SPECT: Myocardial perfusion scintigraphy–single positron emission computed tomography
NHS: National Health Service
NICE: National Institute for Health and Care Excellence
PTL: Pre-Test likelihood

## References

[1] Braunwald E, Jones RH, Mark DB, Brown J, Brown L, Cheitlin MD (1994). Diagnosing and managing unstable angina. Circulation.

[2] National Institute for Health and Clinical Excellence (2010). Chest pain of recent onset: assessment and diagnosis of recent onset chest pain or discomfort of suspected cardiac origin. CG95.

[3] Montalescot G, Sechtem U, Achenbach S, Andreotti F, Arden C, Budaj A (2013). 2013 ESC guidelines on the management of stable coronary artery disease. Eur Heart J.

[4] Fihn SD, Gardin JM, Abrams J, Berra K, Blankenship JC, Dallas AP (2012). 2012 ACCF/AHA/ACP/AATS/PCNA/SCAI/STS guideline for the diagnosis and management of patients with stable ischemic heart disease. J Am Coll Cardiol.

[5] Newby DE, Fox KAA, Flint LL, Boon NA (1998). A ‘same day’ direct-access chest pain clinic: improved management and reduced hospitalization. Q J Med.

[6] Diamond GA, Forrester JS (1979). Analysis of probability as an aid in the clinical diagnosis of coronary-artery disease. N Engl J Med.

[7] Lee AJX, Michael M, Quaderi SA, Richardson JA, Aggarwal SK, Speehly-Dick ME (2015). Implementation of NICE clinical guideline 95 for assessment of stable chest pain in a rapid access chest pain clinical reduces the mean number of investigations and cost per patient. Open Heart.

[8] Genders TS, Steyerberg EW, Alkadhi H, Leschka S, Desbiolles L, Nieman K (2011). A clinical prediction rule for the diagnosis of coronary artery disease: validation, updating, and extension. Eur Heart J.

[9] Shaw LJ, Raggi P, Schisterman E, Berman DS, Callister TQ (2003). Prognostic value of cardiac risk factors and coronary artery calcium screening for all-cause mortality. Radiology.

[10] Patterson C, Nair A, Ahmed N, Bryan L, Bell D, Nicol E (2015). Clinical outcomes when applying the NICE guidance for the assessment of recent-onset chest pain to a rapid access clinic population. Heart.

[11] Meijboom WB, van Mieghem CA, Mollet NR, Pugliese F, Weustink AC, van Pelt N (2007). 64-slice computed tomography coronary angiography in patients with high, intermediate, or low pretest probability of significant coronary artery disease. J Am Coll Cardiol.

[12] Fleischmann KW, Hunink MG, Kuntz KM, Douglas PS (1998). Exercise echocardiography or exercise SPECT imaging? A meta-analysis of diagnostic test performance. JAMA.

[13] Sekhri N, Feder GS, Juhghans C, Hemingway H, Timmis AD (2007). How effective are rapid access chest pain clinics? Prognosis of incident angina and non-cardiac chest pain in 8762 consecutive patients. Heart.

[14] National Institute for Health and Clinical Excellence (2016). Chest pain of recent onset: assessment and diagnosis of recent onset chest pain or discomfort of suspected cardiac origin (update). CG95.

[15] Skinner JS, Smeeth L, Kendall JM, Adams PC, Timmis A (2010). NICE guidance. Chest pain of recent onset: assessment and diagnosis of recent onset chest pain or discomfort of suspected cardiac origin. Heart.

[16] Maddox TM, Stanislawski MA, Grunwald GK, Bradley SM, Ho M, Tsai TT (2014). Nonobstructive coronary artery disease and risk of myocardial infarction. JAMA.

[17] Hernandez R, Vale L (2007). The value of myocardial perfusion scintigraphy in the diagnosis and management of angina and myocardial infarction: a probabilistic economic analysis. Med Decis Mak.

[18] Genders TS, Petersen SE, Pugliese F, Dastidar AG, Fleischmann KE, Nieman K (2015). The optimal imaging strategy for patients with stable chest pain: a cost-effectiveness analysis. Ann Intern Med.

[19] Putting NICE Guidance into Practice. Resource impact report: chest pain of recent onset: assessment and diagnosis. Available at https://www.nice.org.uk/guidance/cg95/resources/resource-impact-report-2726121709 accessed 10 Jan 2017

[20] Mark DB, Federspiel JJ, Cowper PA, Anstrom KJ, Hoffmann U, Patel MR (2016). Economic outcomes with anatomical versus functional diagnostic testing for coronary artery disease. Ann Intern Med.

[21] Greenwood JP, Ripley DP, Berry C, McCann GP, Plein S, Bucciarelli-Ducci C (2016). Effect of care guided by cardiovascular magnetic resonance, myocardial perfusion scintigraphy, or NICE guidelines on subsequent unnecessary angiography rates: the CE-MARC 2 randomized clinical trial. JAMA.

[22] Douglas PS, Hoffman U, Patel MR, Mark DB, Al-Khalidi HR, Cavanaugh B (2015). Outcomes of anatomical versus functional testing for coronary artery disease. N Engl J Med.

[23] Patel MR, Peterson ED, Dai D, Brennan JM, Redberg RF, Anderson HV, Brindis RG, Douglas PS (2010). Low diagnostic yield of elective coronary angiography. N Engl J Med.

[24] Patel MR, Dai D, Hernandez AF, Douglas PS, Messenger J, Garratt KN, Maddox TM, Peterson ED, Roe MT (2014). Prevalence and predictors of nonobstructive coronary artery disease identified with coronary angiography in contemporary clinical practice. Am Heart J.

[25] SCOT-HEART investigators (2015). CT coronary angiography in patients with suspected angina due to coronary artery disease (SCOT-HEART): an open-label, parallel group multicentre trial. Lancet.

[26] Williams MC, Hunter A, Shah AS, Assi V, Lewis S, Smith J (2016). Use of coronary computed tomographic angiography to guide management of patients with coronary disease. J Am Coll Cardiol.

[27] Chow BJ, Small G, Yam Y, Chen L, McPherson R, Achenbach S (2015). Prognostic and therapeutic implications of statin and aspirin therapy in individuals with non-obstructive coronary artery disease: results from the CONFIRM (coronary CT angiography evaluation for clinical outcomes: an international multicenter registry) registry. Arterioscler Thromb Vasc Biol.

[28] Nicol E, Padley S, Roditi G and Roobottom C on behalf of the British Society of Cardiovascular Imaging/ British Society of Cardiovascular Computed Tomography. The challenge of national CT coronary angiography (CTCA) provision in response to NICE CG95 update 2016.

[29] https://www.oecd.org/els/health-statistics-2014-frequently-requested-data.htm

[30] Harden SP, Bull RK, Bury RW, Castellano EA, Clayton B, Hamilton MC (2016). The safe practice of CT coronary angiography in adult patients in UK imaging departments. Clin Radiol.

[31] Abbara S, Blanke P, Maroules CD, Cheezum M, Choi AD, Han BK (2016). SCCT guidelines for the performance and acquisition of coronary computed tomographic angiography: a report of the Society of Cardiovascular Computed Tomography Guidelines Committee endorsed by the north American Society for Cardiovascular Imaging (NASCI). J Cardiovasc Comput Tomogr..

[32] Cury RC, Abbara S, Achenbach S, Agatston A, Berman DS, Budoff MJ (2016). CAD-RADS™ coronary artery disease—reporting and data system. An expert consensus document of the Society of Cardiovascular Computed Tomography (SCCT), the American College of Radiology (ACR) and the north American Society for Cardiovascular Imaging (NASCI). Endorsed by the American College of Cardiology. J Cardiovasc Comput Tomogr.

[33] Arbab-Zadeh A, Fuster V (2016). The risk continuum of atherosclerosis and its implications for defining CHD by coronary angiography. J Am Coll Cardiol.

[34] Ahmadi A, Leipsic J, Blankstein R, Taylor C, Hect H, Stone GW (2015). Do plaques rapidly progress prior to myocardial infarction?. Circ Res.

[35] Pepine CJ, Ferdinand KC, Shaw LJ, Light-McGroary KA, Shah RU, Gulati M (2015). Emergence of nonobstructive coronary artery disease: a woman’s problem and need for change in definition on angiography. J Am Coll Cardiol.

